# An observational study on the effect of seasonal variation on peritoneal dialysis patients

**DOI:** 10.3389/fphys.2023.1172308

**Published:** 2023-07-27

**Authors:** Zanzhe Yu, Li Ding, Yanna He, Jiaying Huang, Wei Fang, Leyi Gu, Zhaohui Ni, Qin Wang

**Affiliations:** Department of Nephrology, Molecular Cell Lab for Kidney Disease, Shanghai Peritoneal Dialysis Research Center, Renji Hospital, Uremia Diagnosis and Treatment Center, Shanghai Jiao Tong University School of Medicine, Shanghai, China

**Keywords:** weather temperature, ultrafiltration, protein intake, fluid management, dialysate glucose concentration

## Abstract

**Background:** Seasonal variation has an impact on plants, wild animals, and also human beings. Data have shown seasonal variation has a significant impact on patients’ fluid status, biochemistry results, and outcomes in hemodialysis populations. The relevant data on peritoneal dialysis is scant.

**Methods:** This was a cross sectional study. All patients followed up in our center had a peritoneal equilibration test and PD adequacy test every 6 months. All the peritoneal equilibration test and PD adequacy test data were collected during December 2019 to November 2020. The monthly delivery information of the whole center was collected from 2015 to 2019.

**Results:** There were 366 patients and 604 sets of peritoneal equilibration test and PD adequacy test results in the study. Plasma albumin and phosphate levels were higher in summer. The monthly average outdoor temperature was positively correlated with plasma albumin. There was no seasonal difference in peritoneal dialysis ultrafiltration or urine volume. The percentage of low glucose concentration (1.5%) usage was higher in summer and lower in winter.

**Conclusion:** Plasma albumin and phosphate levels were higher in summer in PD patients. Weaker glucose peritoneal dialysis dialysate was more widely used in summer. Understanding the seasonal variation of peritoneal dialysis is helpful in individualized treatment.

## Background

Seasonal variation has a wide impact on human beings. It is well known that the mortality rate varies between different seasons. The dominant cause of death is also different between seasons (Keatinge and Donaldson-The Eurowinter Group, 1997). Seasonal effects also vary between different geographical regions. The reasons for seasonal effects are complicated and likely to be a combination of a wide range of issues. Apart from temperature, humidity, and sunlight hours, seasons may also have an impact on food choice and the duration of physical activity (Shephard and Aoyagi, 2009).

For dialysis patients, there is the unique difficulty of maintaining fluid balance. The seasonal effects are likely to be more significant compared with the general population. Studies have shown significant seasonal variation in mortality rates, pre-dialysis systolic blood pressure, intra-dialysis body weight gain, plasma albumin, and CRP levels in hemodialysis patients (Usvyat et al., 2012; Guinsburg et al., 2015). This phenomenon was first recognized in the early 21st century and further described in single-center, multi-center, and registry data. The seasonal effect on blood pressure is highly consistent among studies. Blood pressure is higher in winter (Sposito et al., 2000; Argiles et al., 2004; Broers et al., 2015). In the MONDO registry data, pre-dialysis blood pressure and intra-dialysis body weight gain were lower in summer, and plasma albumin and CRP levels were higher in winter (Guinsburg et al., 2015). The European data, which was also in the MONDO database, showed that sodium, hemoglobin, and CRP were higher in winter, plasma albumin was lower in autumn, and nPCR was lower in winter (Broers et al., 2015). The bioimpedance results showed that overhydration was more significant in summer compared with winter (Broers et al., 2015).

There is limited data on peritoneal dialysis patients regarding seasonal effects. A single-center study from Taiwan showed seasonal variations in peritoneal dialysis. Plasma sodium, potassium, and albumin were lower in summer, and ultrafiltration in PD was also lower in summer (Chen et al., 2010). The same team further noticed a seasonal variation in dialysate glucose concentration usage. Higher glucose concentration dialysate was less used in summer (Li et al., 2008). A single-center study from Beijing showed seasonal variations in blood pressure, but no change in body composition. The study did not show ultrafiltration, urine volume, or dialysate glucose usage (Cheng et al., 2006).

The current study meant to clarify the seasonal effects on peritoneal dialysis patients in Shanghai, a modern city with a typical maritime climate with four distinct seasons. We observed the seasonal variations of biochemical results, membrane function, ultrafiltration volume, and dialysate glucose usage.

## Methods

### Study design and patient population

All patients followed up in our center underwent a first PET test and dialysis adequacy test between 4 and 6 weeks after PD started and then every 6 months. Extra PET tests and dialysis adequacy tests were done if clinically relevant, e.g., unexpected reductions of ultrafiltration or clinical manifestations. All the patients who underwent PET tests and dialysis adequacy tests between December 2019 and November 2020 and who gave consent were enrolled in the study. Only the baseline and 6-month test data were analyzed. The biochemical results, membrane function, and dialysis adequacy data were recorded. In total, 366 patients underwent at least one PET test and 604 sets of data were included in the study.

### Volume management policy of the center

The patients came to their outpatient clinic monthly. The patient’s fluid status was evaluated through their blood pressure, body weight change, and edema status. Bioimpedance, BNP, and other relevant tests were conducted if needed. For patients with fluid overload, furosemide would be increased to 100 mg qd for patients with a urine volume of more than 200 mL per day, before increasing the glucose concentration in the dialysate prescription. For those with daily fluid removal of more than 800 mL per day (urine volume and dialysate ultrafiltration), fluid restriction education would be repeated and emphasized. Their prescribed dialysate regimes may have been change during the clinic visits and then the relevant dialysate shipment was arranged.

### Data collection

The clinical data were collected from the clinical electronic records system. Historical temperature date was retrieved from www.weather.com.cn. The shipment information including dialysate glucose concentration was recorded. To show the seasonal variations of the cohort’s dialysate glucose consumption, the monthly shipment records from 2015 to 2019 were analyzed in the study.

### Statistical analysis

Numerical variables that followed normal distribution were described as mean ± SD. Numerical variables that did not follow normal distribution were described as median (interquartile range). The correlation between the monthly average temperature and other parameters were identified using Pearson’s correlation. The difference between seasons were estimated using one-way ANOVA. IBM SPSS statistics 20 was the software used for the study.

## Results

### Seasonal variation and biochemical profiles

For analyzing purposes, March, April, and May were defined as spring; June, July, and August were defined as summer; September, October, and November were defined as autumn; and December, January, and February were defined as winter.

The seasonal variations of the biochemical profiles are shown in Table 1. Plasma albumin was higher in summer. Phosphate levels were also higher in summer. Hemoglobin levels were lower in spring compared with autumn. There was a positive correlation between the monthly average temperature and plasma albumin. The correlation between phosphate was nearly statistically significant (*r* = 0.075 *p* = 0.066). (Table 2).

**TABLE 1:** Description of seasonal variations in biochemical profiles.

	Spring	Summer	Autumn	Winter	*p*
Albumin (g/L)	37.99 ± 4.33	39.26 ± 4.33	38.13 ± 4.34	37.57 ± 4.24	0.008**
Ca++ (mmol/L)	2.26 ± 0.22	2.25 ± 0.20	2.26 ± 0.21	2.24 ± 0.22	0.904
*p* (mmol/L)	1.67 ± 0.44	1.78 ± 0.48	1.60 ± 0.48	1.70 ± 0.47	0.010**
Hgb (g/L)	107.69 ± 18.94	111.63 ± 17.86	114.59 ± 17.95	111.50 ± 18.37	0.009**
Sodium (mmol/L)	140.16 ± 3.30	140.84 ± 2.67	140.63 ± 3.58	140.61 ± 3.08	0.259
hsCRP (mg/L)	2.2 (0.6,7.8)	2.0 (0.8,5.6)	2.2 (0.8,6.3)	2.7 (1.0,6.4)	0.19
4 h UF (mL)	320 (210,420)	330 (215,440)	300 (185,425)	325 (215,425)	0.377
Daily UF (mL)	498 (223,803)	530 (155,860)	528 (185,815)	373 (63,723)	0.263
Urine volume (mL)	285 (0,900)	300 (0,1000)	250 (0,900)	300 (0,1100)	0.52
D/Pcr4h	0.66 ± 0.10	0.67 ± 0.10	0.66 ± 0.09	0.65 ± 0.11	0.65

*p < 0.01, **p < 0.01.

**TABLE 2:** The correlation between monthly average temperature and biochemical profiles.

	Mean ± SD	Monthly mean maximum temperature		Monthly mean minimum temperature	
		*r*	*p*	*r*	*p*
Age (year)	57.05 ± 15.27	—	—	—	—
Male (%)	60.38	—	—	—	—
Alb (g/L)	38.26 ± 4.35	0.085*	0.039	0.086*	0.036
Ca++ (mmol/L)	2.56 ± 0.21	−0.002	0.955	−0.007	0.861
*p* (mmol/L)	1.69 ± 0.47	0.067	0.1	0.075	0.066
Hgb (g/L)	111.18 ± 18.45	0.014	0.742	0.021	0.608
Sodium (mmol/L)	140.54 ± 3.19	0.034	0.409	0.042	0.303
hsCRP (mg/L)	2.26 (0.77,6.42)	−0.052	0.207	−0.056	0.176

*p < 0.01, **p < 0.01. Lower glucose concentration dialysate in summer.

**FIGURE 1:**
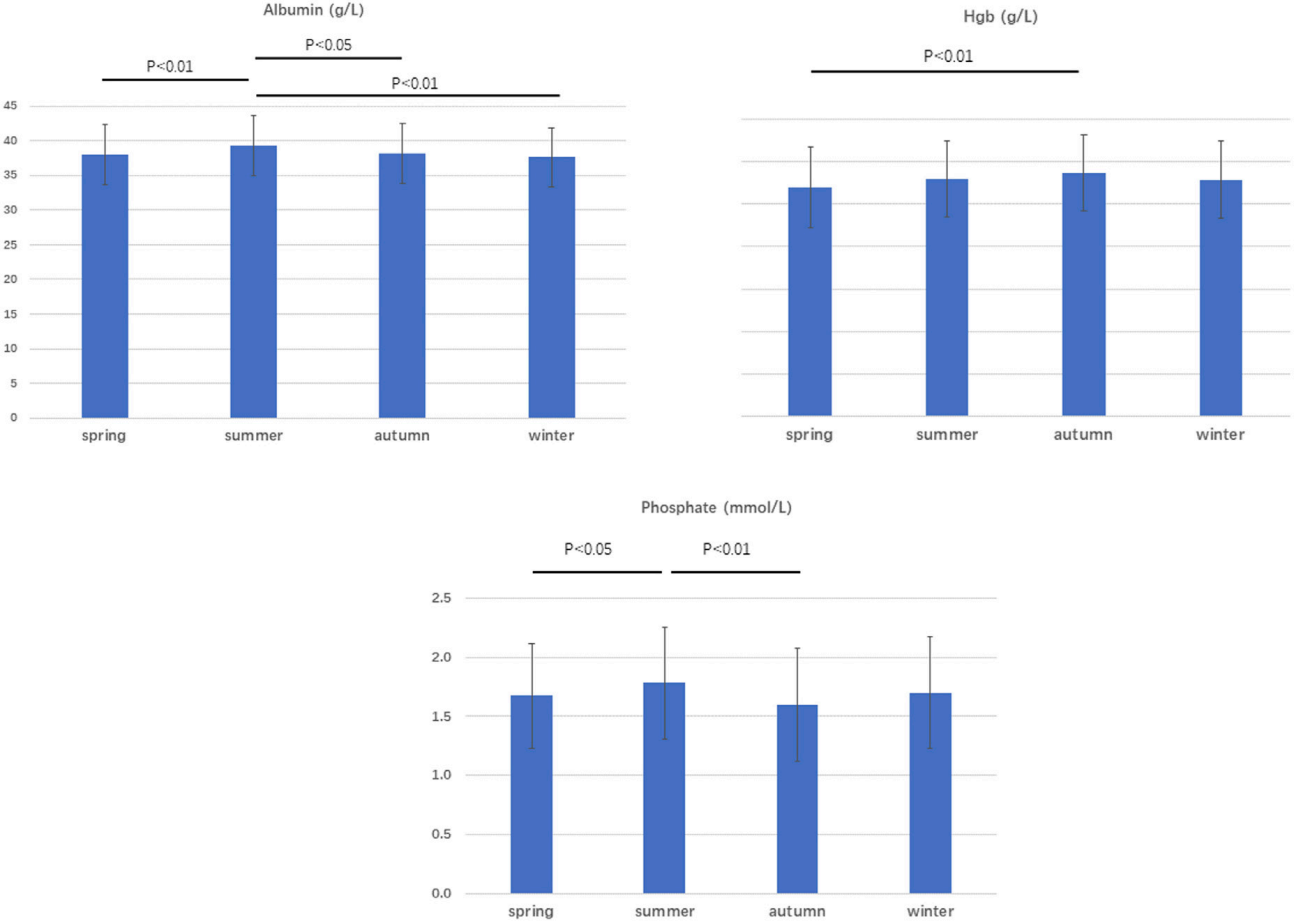
Plasma albumin, phosphate, and Hgb levels according to season.

**FIGURE 2:**
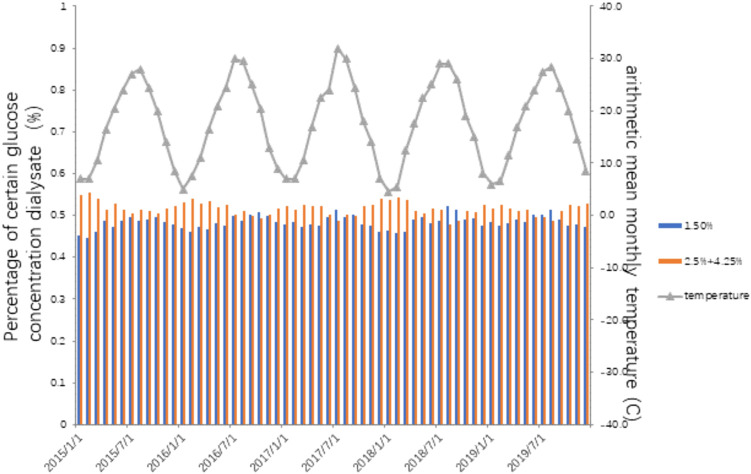
The relationship between monthly average temperature and dialysate glucose concentration.

There was no statistical correlation between the monthly average temperature and daily UF or urine volume (Table 3). As showed in Figures 1, 2, there was a significant seasonal variation in glucose usage. In summer, low glucose concentration dialysate (1.5%) was more widely used than in winter.

**TABLE 3:** The correlation between monthly average temperature and fluid removal.

		Monthly mean maximum temperature		Monthly mean minimum temperature	
		r	*p*	r	*p*
D/Pcr4h	0.66 ± 0.10	0.005	0.903	0.006	0.89
4 h UF (mL)	315 (200,428)	0.016	0.715	0.015	0.731
Daily UF (mL)	495 (158,815)	0.049	0.231	0.054	0.189
Urine volume (mL)	300 (0,1000)	0.045	0.273	0.042	0.309

## Discussion

Seasonal variation has a general impact on plants, animals, and human beings. It has been recognized for a long time. It is not clear how it affects certain populations, in which ways, and to what extent. The current study showed that low glucose concentration dialysate was used in summer. Plasma albumin and phosphate were higher in summer.

### Lower glucose concentration dialysate in summer

We found that lower glucose concentration dialysate was more frequently used in summer. This result is similar to the findings from the Taiwan cohort (Chen et al., 2010). The authors also found lower plasma sodium levels and less daily UF in summer. We did not find this change in our cohort. Extra sodium loss through sweat may be the reason for the low sodium levels in summer. Fluid loss from sweat is also a pathway for fluid removal in dialysis patients. We did not collect blood pressure data in the study. From the study in hemodialysis, blood pressure in summer was lower than in winter. Extra fluid and sodium loss from sweat may have a significant effect on ESRD patients.

### Higher plasma albumin and phosphate in summer

The peak of albumin levels has appeared in different seasons in different studies. In the current study, plasma albumin was higher in summer. In the Taiwan cohort, plasma albumin was negatively correlated with outdoor temperature (Li et al., 2008). In the MONDO database, the European data showed plasma sodium, hemoglobin, and CRP were higher in winter. Plasma albumin was lower in autumn and higher in spring (Broers et al., 2015). In the MONDO registry system, plasma albumin was higher in winter and CRP was also higher in winter (Guinsburg et al., 2015). In the current study, CRP levels were lower in summer and there was a negative correlation between plasma albumin and CRP. The higher albumin level may be a result of less inflammation in summer.

On the other hand, plasma albumin also reflects nutritional status to some degree. Similar to the current study, no studies with a similar focus has detailed food intake data available. It is difficult to get direct evidence to show the relationship between plasma albumin and food intake in different seasons. We also found the phosphate levels were higher in summer. This may reflect a higher protein intake in summer. The seasonal effect on food intake is complicated. Major holidays may relate to high protein intake. Furthermore, the type of food available may be different in different seasons. Weather temperature may also have impact on appetite. In all, the seasonal effect may be different between cultures and geographical locations.

### Improving patient management

Seasonal changes affect the human body in many ways. The reactions vary among different disease situations. Understanding the specific effects of seasonal changes on peritoneal dialysis patients, who have special difficulties in fluid balance, can help improve patient management and patient outcomes. In summer, considering the additional sweat loss of patients, relevant prescription changes can avoid volume deficiency and the related loss of residual renal function.

### Relevance in clinical trials

Seasonal variations should also be considered when designing clinical trials and when interpreting study results. For clinical studies with long enrollment periods crossing different seasons, block randomization should be considered to avoid the impact of seasonal variation on the results (Lin et al., 2015). A balanced number of patients can be enrolled into different groups within short time periods. For clinical studies with observational periods across different seasons, the interpretation of specific variables should fully consider the impact of seasonal variation itself to avoid bias.

## Data Availability

The raw data supporting the conclusion of this article will be made available by the authors, without undue reservation.
